# 1022. Case Study. Burned Out: Therapeutic Approaches to Help with Psychological Distress in Burn ICU Patients

**DOI:** 10.1093/jbcr/irag033.422

**Published:** 2026-04-06

**Authors:** Felix Vazquez, Annie Mun

**Affiliations:** Jacobi Burn Center, Bronx, NY; Jacobi Medical Center, Flushing, NY

## Abstract

**Patient Presentation (age range, injury details, relevant history):**

48 year old Male presenting with 30% TBSA mixed 2nd and 3rd degree partial thickness burns to the head, bilateral upper extremities, upper torso, back, and left hip. Relevant history includes history of delirium, end stage renal disease, exposure keratopathy, and adjustment disorder with depressed mood.

**Clinical Challenges:**

Depressive symptoms affect up to 45% of burn survivors, highlighting the high prevalence of psychological distress in this population. Adverse psychological effects, such as depression secondary to immobilization, ventilator dependence, and metabolic demands of hypermetabolism, can significantly impair mental and physical recovery.

**Management Approach:**

Through the implementation of therapeutic communication and touch, symptoms of depression experienced by long-term burn patients may be alleviated.

Therapeutic Touch: Application of ointments with extensive massage and stretching of healed and at-risk-of-hypertrophy graft sites to prevent contracture, and to provide comfort and rest.

Therapeutic Communication: Empathetic, patient-centered dialogue incorporating humor to reduce stress, encourage involvement in care, and restore daily rhythm.

**Outcomes:**

After implementing regular therapeutic touch and communication, the patient demonstrated measurable improvements across psychological and functional domains. Depressive symptoms, using PHQ-9 criteria, appeared to decrease from moderate to mild levels after these interventions. The patient exhibited increased engagement in activities of daily living, reflected in better circadian rhythm with reduced signs of delirium, consistent with CAM-ICU assessments. Sleep quality, inferred through patient-reported restfulness, improved concurrently with mood. Improvements were most pronounced during periods of consistent intervention and declined when interventions were interrupted, underscoring their direct impact on patient well-being.

**Lessons Learned:**

For 3-5 months, the patient received medical care with limited emotional support. After healthcare staff incorporated psychological support into their daily routines, a clear difference in the patients' PHQ-9 scoring and CAM-ICU correlated with the desire to improve. Improvements were consistent with interventions and declined in their absence, highlighting the effectiveness of these interventions.

**Applicability to Practice:**

This case illustrates the broader applicability of integrating psychosocial support into routine burn care. Patients with extensive burns often experience a cascade of adverse effects—hypermetabolism, muscle wasting, fatigue, diminished participation, and lowered self-worth—exacerbated by depression and anxiety. It's not only a physical injury but a psychological one that requires as much as an open wound. Structured interventions, such as therapeutic touch and patient-centered communication, can help disrupt this cycle, leading to improved emotional and functional outcomes.

